# Rhizodeposition of Nitrogen and Carbon by Mungbean (*Vigna radiata* L.) and Its Contribution to Intercropped Oats (*Avena nuda* L.)

**DOI:** 10.1371/journal.pone.0121132

**Published:** 2015-03-30

**Authors:** Huadong Zang, Xuechao Yang, Xiaomin Feng, Xin Qian, Yuegao Hu, Changzhong Ren, Zhaohai Zeng

**Affiliations:** 1 College of Agronomy and Biotechnology, China Agricultural University, Beijing, China; 2 Baicheng Academy of Agricultural Sciences, Baicheng, Jilin, China; Northwest A&F University, CHINA

## Abstract

Compounds released by mungbean roots potentially represent an enormous source of nitrogen (N) and carbon (C) in mungbean-oat intercropping systems. In this study, an *in situ* experiment was conducted using a ^15^N - ^13^C double stem-feeding method to measure N and C derived from the rhizodeposition (NdfR and CdfR) of mungbean and their transfer to oats in an intercropping system. Mungbean plants were sole cropped (S) or intercropped (I) with oat. The plants were labeled 5 weeks after planting and were harvested at the beginning of pod setting (I_p_ and S_p_) and at maturity (I_m_ and S_m_). More than 60% and 50% of the applied ^15^N and ^13^C, respectively, were recovered in each treatment, with ^15^N and ^13^C being quite uniformly distributed in the different plant parts. NdfR represented 9.8% (S_p_), 9.2% (I_p_), 20.1% (S_m_), and 21.2% (I_m_) of total mungbean plant N, whereas CdfR represented 13.3% (S_p_), 42.0% (I_p_), 15.4% (S_m_), and 22.6% (I_m_) of total mungbean plant C. When considering the part of rhizodeposition transferred to associated oat, intercropping mungbean released more NdfR and CdfR than mungbean alone. About 53.4–83.2% of below-ground plant N (BGP-N) and 58.4–85.9% of BGP-C originated from NdfR and CdfR, respectively. The N in oats derived from mungbean increased from 7.6% at the pod setting stage to 9.7% at maturity, whereas the C in oats increased from 16.2% to 22.0%, respectively. Only a small percentage of rhizodeposition from mungbean was transferred to oats in the intercropping systems, with a large percentage remaining in the soil. This result indicates that mungbean rhizodeposition might contribute to higher N and C availability in the soil for subsequent crops.

## Introduction

Legume-cereal intercropping is an agronomic technique that is known to potentially improve crop yield by increasing resource use efficiency, and has been widely and consistently used in China. This cropping systems has facilitated sustainable and organic food-production systems [1, 2]. Most existing studies on legume-cereal intercropping systems have focused on quantifying the amount of N_2_ fixation and nitrogen (N) transfer and acquisition [3]. However, information about the overall turnover of rhizodeposition in the intercropping systems and carbon (C) transfer from legumes to cereals remains limited.

Rhizodeposition is defined as all root-derived compounds and plant materials that are released from living roots during plant growth [4, 5]. A wide range of organic compounds are involved in this process [6, 7], including inorganic ions, sloughed cells, and root hairs [8]. Thus, rhizodeposition has diverse functions in plant nutrition and soil ecology, such as improving nutrient availability, acting as allelochemicals, and serving as a carbon and energy source for rhizosphere soil microorganisms [9]. Previous studies on rhizodeposition have focused on mono-cropping systems and were generally performed under greenhouse or laboratory conditions. Different ^15^N-labeling techniques have been used to measure the amount of N rhizodeposition, including ^15^N leaf feeding [10–12], ^15^N petiole feeding [13, 14], ^15^N stem injection [15], ^15^N_2_ and ^15^NH_3_ atmospheric labeling [16–18], and the split-root technique [19–22]. To estimate the rhizodeposition of C, photoassimilates labeled with ^14^CO_2_ or ^13^CO_2_ have been used to trace the flow of C into the soil and to monitor its further transformation [23–27]. Few studies have used both C and N tracers to investigate rhizodeposition, particularly under field conditions [28, 29]. Russell and Fillery [30] developed an in *situ* stem labeling method, in which an isotope solution is taken up by the whole plant via a cotton wick that passes through the plant stem. This method has been previously applied for the ^13^C-^15^N double labeling of peas and oats by using a glucose-urea mixed solution [29, 31]. Unlike other *in situ* shoot feeding techniques [32, 33], this method facilitates relatively uniform isotope enrichment and high total recovery rates [34]. In addition, this technique does not cause root damage, unlike the split-root method [35], making it appropriate for C and N rhizodeposition research under field conditions.

It has been reported that legume rhizodeposition N at maturity varies from 4% to 71.1% of total plant N [13, 19, 21–22, 29–31], and is higher compared with non-legumes, because of N_2_-fixation [29]. Khan et al. [13] showed that N rhizodeposition by mungbean represents 17% of total plant N. Below-ground N transfer from the cowpea to millet in intercropping was demonstrated under field conditions, with significant levels of approx. 10 kg N ha^-1^ [33]. In addition, 19% of N in barley acquired from intercropped peas was also reported [20]. Existing studies show that about 40% of the net fixed C is allocated below ground [36], while around 11% is retained in rhizodepostion [37]. More than 20% of the total assimilated plant C is released from roots via rhizodeposition during the vegetative period of different plant species [9]. About one-third of the below-ground carbon becomes CO_2_ by root respiration and microbial utilization [36]. The remaining part of below-ground translocated C may be incorporated into the soil organic matter or microbial biomass. However, this information was obtained from sole cropping systems. Our understanding of the part of C transferred to crops in intercropping systems remains limited.

Mungbean (*Vigna radiata* L.) is an important food and economic crop throughout China. This crop grows over a wide range of agro-climatic zones in the country. Baicheng is the largest mungbean producing area in northeastern China. Intercropping mungbean with oats (*Avena nuda* L.) is recommended in this area to control wind-driven soil erosion and to increase productivity [38, 39]. However, our understanding of mungbean rhizodeposition in the soil and its transfer to intercropped oats under field conditions remains limited.

The objectives of this study were to (1) quantify N and C rhizodeposition, uptake, and distribution in the different plant parts of mungbean and oats under field conditions by using the stem double-labeling method, (2) quantify N and C transfer from mungbean to oats in an intercropping system, and (3) compare N and C rhizodeposition of mungbean and their transfer to intercropped oats at different growth stages.

## Materials and Methods

### Study site

**Fig 1:**
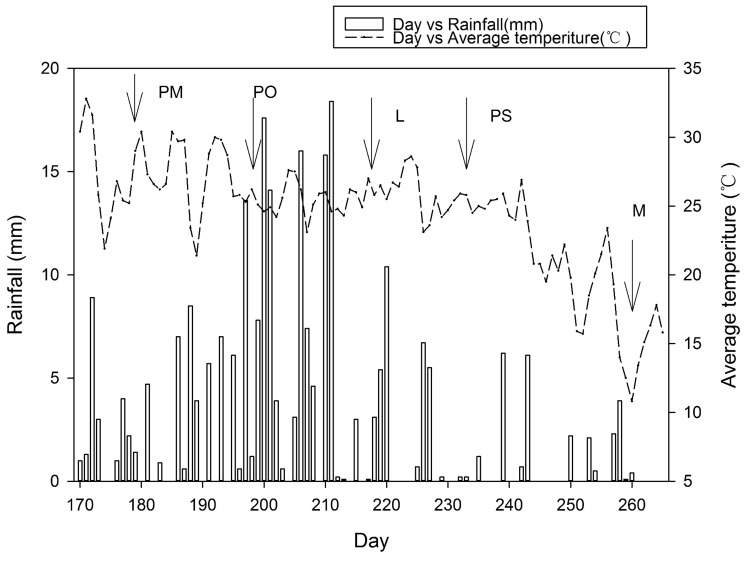
Daily rainfall and average temperature during the experimental period (2011). The dots represent the different activities. PM: plant mungbean, PO: plant oat, L: labelling, PS: mungbean at pod setting, M: mungbean at maturity.

The experiment was conducted at the farm of Baicheng Academy of Agricultural Sciences, Baicheng, Jilin, China (45° 37′N, 122° 48′E, 152 m above sea level). The local climate is temperate continental monsoon, with an average annual rainfall of 407.9 mm. During the experimental period in 2011, precipitation was 231.4 mm, and the average temperature was 20.2°C. The soil is a chernozem and was collected from a field that had been fallow for 5 years. The soil chemical properties, rainfall, and temperature at the site between June and September 2011 are shown in Table 1 and Fig. 1, respectively.

**Table 1:** Soil chemical properties, sampled at 0–30 cm depth.

Soil parameters	
pH (H_2_O)	7.2
Organic matter (g kg^-1^)	14.7
Total N (g kg^-1^)	0.8
Available N (mg kg^-1^)	59.6
Available P (mg kg^-1^)	9.9
Available K (mg kg^-1^)	76.6

The methodology of soil analysis used were: pH (potentiometric analysis), Organic matter (potassium bichromate titrimetric method), Total N (the Kjeldahl), Available N (Alkaline hydrolysis diffusion), Available P (Mo-Sb colorimetric method), Available K (flame photometric meter method)

### Experimental design and ^13^C-^15^N double labeling

In the spring of 2011, 16 polyvinyl chloride plastic (PVC) columns (diameter, 20 cm; length, 55 cm) with a plastic pallet (diameter, 25 cm) at the bottom were buried in the field. The surface soil (0–30 cm) from the field was collected, air-dried, and passed through a 3 mm sieve to remove gravel and plant residue. About 19 kg of the dry soil was placed in each column and watered daily to maintain a water holding capacity of between 40 and 80%.

**Fig 2:**
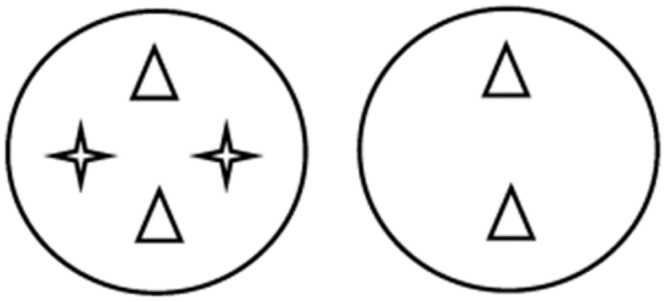
How the crops were planted in the plastic (PVC) columns for solo and intercropping treatment. The triangles stand for mungbeans and the quadrangles stand for oats.

The experiment was established in a RCBD (randomized complete block design) with 4 replicates for each treatment. Ten columns were used for intercropping of mungbean with oats, of which 8 were used for the label treatment and 2 for the control. The other 10 columns were used for sole mungbean cropping, of which 8 were used for the label treatment and 2 for the control. Six oat and 6 mungbean seeds were sown in each intercropped column and thinned to 2 oat and 2 mungbean seedlings after emergence. The intercropping treatment was planted as shown in Fig. 2. Six mungbean seeds were sown in the mono-cropped columns and thinned to 2 plants per column after emergence. Corresponding to each ^15^N and ^13^C labeling treatment, an unlabeled control treatment was planted and harvested the same as the pot treatment to obtain background values of ^15^N and ^13^C for the different plant and soil parts (as show in Fig. 2).

The mungbean variety used in this experiment was Bailv 11, which matures in about 100 days, while the oat variety was Baiyan 2, which matures in about 85 days. Both varieties were provided by Baicheng Academy of Agricultural Sciences. Mungbean was planted on June 29 after accelerated germination, whereas the oats were planted 20 days later. This planting technique is the normal practice in the study area.

The isotope feeding method used in this experiment was the ^15^N-^13^C double-labeling method [29, 34, 40]. In brief, a needle was used to pass a cotton wick through the stem at approximately 3 cm above the soil surface. The end of the wick was inserted into a 1.5-mL liquid chromatography injection bottle that contained the labeling liquid. Transpiration was prevented by covering the wick with a soft plastic tube, except for the part that was inserted into the stem. The labeling solution was 0.2% (w/v) ^15^N enriched (99 atom%) urea and 4% (w/v) ^13^C enriched (99 atom%) glucose. The solutions were prepared separately using sterile deionized water under aseptic conditions at twice the target concentration, and were mixed together in the injection bottle when the labeling system was set. All of the materials used in the system were sterilized for 20 min at 121°C.

Measurements were performed in 2 stages; namely, mungbean pod setting and maturation. Therefore, a total of 4 measurements were performed when combined with the cropping methods; namely, S_p_ = mungbean harvested at pod setting; S_m_ = mungbean harvested at maturity; I_p_ = intercropping system harvested at mungbean pod setting; and I_m_ = intercropping system harvested at mungbean maturity. S_p_, I_p_, and the respective controls were harvested at the onset of mungbean pod setting (August 24, 2011), whereas S_m_, I_m_, and the respective controls were harvested at mungbean maturity (September 19, 2011).

Mungbean was labeled twice at weekly intervals starting on the fifth week after planting (August 7, 2011). For each labeling, 1 mL labeling solution was used. Furthermore, absorption of all the solute by the plant was ensured by adding a further 1 mL of deionized water twice after the solution had been taken up by the plants. The vials were placed for 24 h and 48 h (depending on the speed of uptake) and were removed after the completion of labeling. After labeling, a 1-mm mesh was placed on the ground around the labeled plants to collect any fallen leaves and to prevent the soil from being enriched by plant material contaminants.

### Plant and soil sampling

During sample collection, the whole of the above-ground plants (AGP) were directly cut above the soil surface. Mungbean was separated into the leaves, stems, and pods, while the AGP of oats was sampled as a single plant part. The soil and roots were also sampled at the same time. First, the columns were removed from the field, the top of each column was sealed with a plastic bag, and the soil from each column was weighed and placed on a plastic sheet. Subsequently, the soil was passed through a 2-mm sieve, and all visible roots were collected manually. The sieved soil was thoroughly mixed, and a 300-g soil sample was obtained for analysis. A sub-sample of 100 g was dried at 60°C for at least 72 h to determine the weight, and ^13^C and ^15^N contents. The other 200 g was washed using a 200-μm sieve to determine the amount of remaining rootlets. After the samples were dried at 60°C for at least 72 h, the weight was determined. The samples were then ground to a fine powder using a centrifuge mill and a ball mill, the total N and C content and ^15^N and ^13^C enrichment in the plant and soil samples were determined using an isotope ratio mass spectrometer (Vario EL, Elementar, Germany) coupled with a Vario PYRO Cube Elemental analyzer. Plant samples were dried at 105°C for 1.5 h and at 60°C to constant weight, the dry weight was determined.

### Calculations and statistical analysis

The percentage of soil N and C derived from rhizodeposition (%NdfR and %CdfR, respectively) was calculated using the following equations [41]:

$$\text{\%NdfR}=\frac{{\text{atom\% }}^{15}{\text{N Soil }}_{\text{Labeled}}{\text{ - atom\% }}^{15}{\text{N Soil }}_{\text{Control}}}{{\text{atom\% }}^{15}{\text{N Root }}_{\text{Labeled}}{\text{ - atom\% }}^{15}{\text{N Root }}_{\text{Control}}}\times 100$$ (1)

%CdfR=atom% 13C  Soil Labeled - atom% 13C  Soil Controlatom% 13C Root Labeled - atom% 13C Root Control×100 (2)

The quantities of NdfR and CdfR were calculated as follows [29, 34]:

$$\text{NdfR}={\text{Total N}}_{\text{soil}}\times \text{\%NdfR}$$ (3)

$$\text{CdfR}={\text{Total C}}_{\text{soil}}\times \text{\%CdfR}$$ (4)

N and C transfer from the labeled mungbean to oats was measured by comparing ^15^N and ^13^C atoms in the mungbean roots and in the receiver’s (oat) biomass. The control was the reference unlabeled crop. The proportion of N and C in the oat AGP and roots was calculated using the following equation [33]:

$$\%\text{Ntransfer}=\frac{{\text{atom\% }}^{15}{\text{N Receiver - atom\% }}^{15}\text{N Control}}{{\text{atom\% }}^{15}{\text{N Root }}_{\text{Labeled}}{\text{ - atom\% }}^{15}{\text{N Root }}_{\text{Control}}}\times 100$$ (5)

$$\%\text{Ctransfer}=\frac{{\text{atom\% }}^{\text{13}}{\text{C Receiver - atom\% }}^{\text{13}}\text{C Control}}{{\text{atom\% }}^{\text{13}}{\text{C Root }}_{\text{Labeled}}{\text{ - atom\% }}^{\text{13}}{\text{C Root }}_{\text{Control}}}\times 100$$ (6)

In this experiment the receiver means oats and labeled plants were mungbean, while the control means the corresponding unlabeled plants. The quantities of N and C that were transferred to the oats were calculated as follows [33]:

$$\text{Ntransfer}={\text{Total N}}_{\text{Receiver}}\times \%\text{Ntransfer}$$ (7)

$$\text{Ctransfer}={\text{Total C}}_{\text{Receiver}}\times \%\text{Ctransfer}$$ (8)

The average data and errors were calculated by Microsoft Excel (Version 2010; Microsoft Inc., USA), while SigmaPlot (Version 12.5; Systat Software Inc., USA) was used to compile the Fig. 1. The equations were edited by MathType (Version 6.0, Design Science Inc., California, USA). The significant effect was compared using Tukey's HSD (Honestly Significant Difference) test at *p < 0*.*05*. The analyses of variance were performed using JMP (Version 9.0, SAS Institute Inc., USA) to determine the treatment effects.

## Results

### Recovery and distribution of ^15^N and ^13^C

^15^N enrichment was the highest in the stem fraction, followed by the grain, leaves, and roots for mungbean, and was the lowest in the AGP and root fraction of oats. ^15^N enrichment was generally below 0.3 atom% ^15^N excess. However, atom% ^15^N excess of the different plant parts did not show any significant difference between treatments (*p < 0*.*05*). The total recovery of applied ^15^N was between 62% and 82%, and it was slightly higher at maturity than at pod setting, but there was no significant difference between different treatments (*p < 0*.*05*). More than 80% of the recovered isotopes were present in the above-ground parts of mungbean. The amount of ^15^N that was recovered increased from pod setting to maturity in the grains, but decreased in the stems, leaves, and below-ground plant (BGP) fractions over the same period. At the pod setting stage, more than 60% of the recovered ^15^N was detected in the leaves and stems of mungbean. Less ^15^N was recovered in the intercropping system than in the sole cropping system. At maturity, more than half of the recovered ^15^N was detected in mungbean grains, which was 3 times higher than that recorded at pod setting. The amount of ^15^N recovered from rhizodeposition increased over time, with a maximum of 9.82% and 7.06% ^15^N being recovered from S_m_ and I_m_, respectively, with 0.53% of recovered ^15^N being found in the intercropped oats (Table 2).

**Table 2:** Atom% ^15^N excess, recovery and distribution of ^15^N in each part of plant and soil in sole cropped and intercropped systems.

		Mungbean				Oat			
		Grain	Stem	Leaves	Roots	AGP ^1^	Roots	Rhizodeposition	Total
Atom% 13C excess	S_p_	0.04±0.02a	0.25±0.06a	0.08±0.02a	0.08±0.01a	-	-	-	-
	I_p_	0.03±0.00^a^	0.25±0.04^a^	0.05±0.02^a^	0.10±0.01^a^	0.003±0.000^a^	0.02±0.00^a^	-	-
	S_m_	0.02±0.00^a^	0.20±0.04^a^	0.05±0.02^a^	0.12±0.07^a^	-	-	-	-
	I_m_	0.02±0.00^a^	0.19±0.05^a^	0.06±0.02^a^	0.07±0.00^a^	0.001±0.000^b^	0.01±0.00^b^	-	-
Recovery of 13C (in % of applied)	S_p_	1.59±0.07^a^	33.99±4.90a^b^	11.97±2.82^a^	3.52±0.05^a^	-	-	4.82±3.24^a^	55.88±8.23^a^
	I_p_	2.70±1.44^a^	36.47±6.07^a^	8.20±3.37a^b^	5.27±5.45^a^	0.04±0.03^a^	0.08±0.00^a^	14.54±11.35^a^	67.30±11.25^a^
	S_m_	6.88±1.59^a^	22.44±6.00^c^	4.68±1.69^b^	5.46±3.20^a^	-	-	11.08±8.62^a^	50.54±11.91^a^
	I_m_	7.74±5.89^a^	24.80±2.75b^c^	5.72±0.92^b^	2.64±1.32^a^	0.07±0.03^a^	0.11±0.06^a^	11.00±6.13^a^	52.10±7.35^a^
Distribution of recovered 13C (%)	S_p_	2.84±1.07^b^	60.91±3.64^a^	21.25±2.51^a^	6.41±1.30^a^	-	-	8.59±5.11^a^	100
	I_p_	4.18±2.43a^b^	55.27±12.18^a^	12.42±5.42^b^	7.52±6.69^a^	0.06±0.04^a^	0.13±0.02^a^	20.43±16.59^a^	100
	S_m_	14.07±4.03a^b^	45.38±11.69^a^	9.34±2.71^b^	10.96±6.11^a^	-	-	20.24±10.75^a^	100
	I_m_	14.62±9.73^a^	48.47±9.39^a^	11.04±1.38^b^	4.97±2.03^a^	0.15±0.06^a^	0.23±0.16^a^	20.53±9.59^a^	100

^1^ Above-ground part

Mungbean were labeled five weeks after planting and harvested at the beginning of pod setting and at maturity. Mungbean were divided into grain, stem, leaves and root, while oats were divided into above-ground part and root, and rhizodeposition.

The abbreviation in the table represent different treatments. S_p_: sole mungbean harvested at pod setting, S_m_: sole mungbean harvested at maturity, I_p_: intercropping system harvested at mungbean pod setting, and I_m_: intercropping system harvested at mungbean maturity. Values are means±standard error (n = 4). Values with different letters within a column indicate significant differences between the treatment S_p_, I_p_, S_m_ and I_m_ (Tukey HSD, *p<0*.*05*).

^13^C enrichment was similar to ^15^N enrichment, with no significant difference between the different treatments (*p < 0*.*05*). ^13^C enrichment ranged between 0.001 atom% ^13^C excess and 0.25 atom% ^13^C excess, and was the highest in the mungbean stem fraction. More than 70% of the recovered isotopes were present in the above-ground parts of mungbean. The amount of ^13^C recovered from rhizodeposition in the I_p_ treatment was 20.62% (including the C transferred to oats), which was twice that of the S_p_ treatment. In both the S_m_ and I_m_ treatments, about 20% of the recovered ^13^C was detected in the BGP. The recovered ^13^C in the intercropped oats was 0.19% and 0.38% of the total recovered ^13^C at pod setting and maturity, respectively (Table 3).

**Table 3:** Atom% ^13^C excess, recovery and distribution of ^13^C in each part of plant and soil in sole cropped and intercropped systems.

		Mungbean				Oat			
		Grain	Stem	Leaves	Roots	AGP ^1^	Roots	Rhizodeposition	Total
Atom% ^13^C excess	S_p_	0.04±0.02^a^	0.25±0.06^a^	0.08±0.02^a^	0.08±0.01^a^	-	-	-	-
	I_p_	0.03±0.00^a^	0.25±0.04^a^	0.05±0.02^a^	0.10±0.01^a^	0.003±0.000^a^	0.02±0.00^a^	-	-
	S_m_	0.02±0.00^a^	0.20±0.04^a^	0.05±0.02^a^	0.12±0.07^a^	-	-	-	-
	I_m_	0.02±0.00^a^	0.19±0.05^a^	0.06±0.02^a^	0.07±0.00^a^	0.001±0.000^b^	0.01±0.00^b^	-	-
Recovery of ^13^C (in % of applied)	S_p_	1.59±0.07^a^	33.99±4.90^ab^	11.97±2.82^a^	3.52±0.05^a^	-	-	4.82±3.24^a^	55.88±8.23^a^
	I_p_	2.70±1.44^a^	36.47±6.07^a^	8.20±3.37^ab^	5.27±5.45^a^	0.04±0.03^a^	0.08±0.00^a^	14.54±11.35^a^	67.30±11.25^a^
	S_m_	6.88±1.59^a^	22.44±6.00^c^	4.68±1.69^b^	5.46±3.20^a^	-	-	11.08±8.62^a^	50.54±11.91^a^
	I_m_	7.74±5.89^a^	24.80±2.75^bc^	5.72±0.92^b^	2.64±1.32^a^	0.07±0.03^a^	0.11±0.06^a^	11.00±6.13^a^	52.10±7.35^a^
Distribution of recovered ^13^C (%)	S_p_	2.84±1.07^b^	60.91±3.64^a^	21.25±2.51^a^	6.41±1.30^a^	-	-	8.59±5.11^a^	100
	I_p_	4.18±2.43^ab^	55.27±12.18^a^	12.42±5.42^b^	7.52±6.69^a^	0.06±0.04^a^	0.13±0.02^a^	20.43±16.59^a^	100
	S_m_	14.07±4.03^ab^	45.38±11.69^a^	9.34±2.71^b^	10.96±6.11^a^	-	-	20.24±10.75^a^	100
	I_m_	14.62±9.73^a^	48.47±9.39^a^	11.04±1.38^b^	4.97±2.03^a^	0.15±0.06^a^	0.23±0.16^a^	20.53±9.59^a^	100

^1^ Above-ground part

Mungbean were labeled five weeks after planting and harvested at the beginning of pod setting and at maturity. Mungbean were divided into grain, stem, leaves and root, while oats were divided into above-ground part and root, and rhizodeposition.

The abbreviation in the table represent different treatments. S_p_: sole mungbean harvested at pod setting, S_m_: sole mungbean harvested at maturity, I_p_: intercropping system harvested at mungbean pod setting, and I_m_: intercropping system harvested at mungbean maturity. Values are means±standard error (n = 4). Values with different letters within a column indicate significant differences between the treatment S_p_, I_p_, S_m_ and I_m_ (Tukey HSD, *p<0*.*05*). ^yyyy^

### Quantity and partitioning of N and C

Mungbean dry matter showed no significant difference in the sole or intercropping systems. However, significantly more dry matter was obtained at the mature stage in mungbean grains and leaves (*p < 0*.*05*) (Table 4). At the pod setting stage, NdfR represented 9.8% and 9.2% of the total N, contributing to 57.2% and 53.4% of BGP-N, in the S_p_ and I_p_ treatments, respectively. When combined with the N transferred to the intercropped oat, the total NdfR in I_p_ was 97.0 mg plant^-1^, which was 3.7% higher than that in S_p_. About 40% of the C in I_p_ was detected in the BGP fraction, which was more than fifth that of the corresponding sole cropping treatment. In addition, CdfR was higher than NdfR, representing 13.3% and 42.0% of total C, contributing to 58.4% and 85.9% of BGP-C, in the S_p_ and I_p_ treatments, respectively. After adding the C that was transferred to intercropped oats, the total C derived from rhizodeposition was 10.15 g plant^-1^ in I_p_, which was almost 6 times that of S_p_, but there was no significant difference (*p > 0*.*05*) (Table 5).

**Table 4:** Mungbean dry matter in each part of sole cropped and intercropped plants.

	S_p_	I_p_	S_m_	I_m_
**Mungbean**				
Grain	3.69±2.48 ^b^	5.80±1.82 ^b^	26.44±3.23 ^a^	23.54±3.37 ^a^
Stem	8.18±0.87 ^a^	10.44±3.62 ^a^	10.41±1.51 ^a^	11.01±1.10 ^a^
Leaves	8.01±0.51 ^b^	8.55±2.45 ^b^	11.78±0.50 ^a^	11.88±1.14 ^a^
Roots	3.43±0.39 ^a^	4.26±0.00 ^a^	3.28±0.22 ^a^	3.16±1.15 ^a^

The abbreviation in the table represent different treatments. S_p_: sole mungbean harvested at pod setting, S_m_: sole mungbean harvested at maturity, I_p_: intercropping system harvested at mungbean pod setting, and I_m_: intercropping system harvested at mungbean maturity.

Values are means±standard error (n = 4).Values with different letters within a line indicate significant differences between the means (Tukey HSD, *p <0*.*05*).

**Table 5:** Nitrogen and carbon content in each part of mungbean, and the transfer to rhizodeposition and intercropped oat.

	Nitrogen content (mg plant^-1^)				Carbon content (g plant^-1^)			
	S_p_	I_p_	S_m_	I_m_	S_p_	I_p_	S_m_	I_m_
**Mungbean**								
Grain	145.3±92.2^b^	229.6±70.5^b^	875.5±144.7^a^	823.5±149.3^a^	1.60±1.10^b^	2.59±0.83^b^	11.83±1.46^a^	10.58±1.55^a^
Stem	191.2±28.7^a^	198.0±42.8^a^	104.4±50.1^b^	115.0±47.2^b^	4.35±0.66^a^	4.60±0.45^a^	3.41±0.38^b^	4.39±1.55^b^
Leaves	454.9±42.2^a^	415.1±58.3^a^	171.0±12.4^b^	201.0±82.5^b^	4.98±0.23^a^	5.01±0.48^a^	3.01±0.27^a^	3.37±1.02^a^
Roots	69.7±12.8^a^	79.7±4.0^a^	65.0±6.6^a^	58.5±19.5^a^	1.34±0.11^a^	1.59±0.09^a^	1.36±0.12^a^	1.19±0.36^a^
**Oat**								
AGP^1^	-	0.5±0.4^b^	-	5.5±1.2^a^	-	0.03±0.02^b^	-	0.45±0.38^a^
Roots	-	2.4±1.4^a^	-	7.3±3.7^a^	-	0.06±0.03^a^	-	0.06±0.03^a^
Rhizodeposition	93.5±56.3^a^	94.1±76.4^a^	306.1±365.6^a^	326.4±27.6^a^	1.88±1.36^a^	10.06±12.48^a^	3.58±2.71^a^	6.28±4.80^a^
Total	954.7±232.2^a^	1019.3±253.8^a^	1522.0±579.4^a^	1537.4±331.1^a^	14.14±3.46^a^	24.34±14.59^a^	23.19±4.93^a^	27.83±10.04^a^

^1^ Above-ground part

The data of mungbean in this table means the total N and C content of mungbean. The data of oat in this table means the N and C derived from mungbean. Mungbean were labelled five weeks after planting and harvested at the beginning of pod setting and at maturity. Mungbean were divided into grain, stem, leaves and root, while oats were divided into above-ground part and root, and rhizodeposition.

The abbreviation in the table represent different treatments. S_p_: sole mungbean harvested at pod setting, S_m_: sole mungbean harvested at maturity, I_p_: intercropping system harvested at mungbean pod setting, and I_m_: intercropping system harvested at mungbean maturity. Values are means±standard error (n = 4). Values with different letters within a line indicate significant differences between the treatment S_p_, I_p_, S_m_ and I_m_ (Tukey HSD, *p<0*.*05*).

The NdfR was higher at maturity in both the sole and intercropping treatments, representing 20.1% and 21.2% of total N, and contributing to 82.5% and 83.2% of the BGP-N, respectively. When combined with the amount of N transferred to oats, total NdfR was 10.8% higher in I_m_ compared to S_m_. The C derived from rhizodeposition in the S_m_ and I_m_ treatments represented 15.4% and 22.6% of total recovered C, accounting for 72.8% and 79.2% of BGP-C, respectively (Table 5).

### N and C transfer from mungbean to intercropped oat

In the I_m_ treatment, the N content of the AGP and roots of oats was 131.8 mg plant^-1^ and 20.4 mg plant^-1^, respectively. These values were about 3 times higher than those in the I_p_ treatment. In the AGP of oats, 0.5 and 5.5 mg plant^-1^ N was derived from mungbean in treatments I_p_ and I_m_, which represented 1.5% and 4.6% N content, respectively. In addition, the total N derived from mungbean was significantly higher in treatment I_m_ compared to I_p_ (*p < 0*.*05*). The percentage of N derived from mungbean in oats increased from 7.6% at pod setting to 9.7% at maturity (Table 6).

**Table 6:** N and C content in intercropping oat and derived from mungbean.

	N content in oat (mg plant^-1^)		N derived from mungbean (mg plant^-1^)		N derived from mungbean (%)		C content in oat (mg plant^-1^)		C derived from mungbean (mg plant^-1^)		C derived from mungbean (%)	
	I_p_	I_m_	I_p_	I_m_	I_p_	I_m_	I_p_	I_m_	I_p_	I_m_	I_p_	I_m_
AGP^1^	43.2±21.7^a^	131.8±55.0^a^	0.5±0.4^b^	5.5±1.2^a^	1.5±1.3^a^	4.6±2.1^a^	0.42±0.22^b^	1.73±0.64^a^	0.03±0.02^a^	0.45±0.38^a^	8.2±5.1^a^	24.5±13.1^a^
Root	9.7±0.0^b^	20.4±2.4^a^	2.4±1.4^a^	7.3±3.7^a^	30.7±8.6^a^	37.4±21.2^a^	0.16±0.00^b^	0.51±0.09^a^	0.06±0.03^a^	0.06±0.03^a^	37.4±16.3^a^	12.5±8.3^b^
Total	52.9±21.7^b^	152.3±57.3^b^	2.9±1.3^b^	12.8±3.3^a^	7.6±4.3^a^	9.7±5.2^a^	0.58±0.22^b^	2.24±0.72^a^	0.09±0.04^a^	0.51±0.35^a^	16.2±6.4^a^	22.0±9.5^a^

^1^ Above-ground part

The abbreviation in the table represent different treatments. I_p_: intercropping system harvested at mungbean pod setting, and I_m_: intercropping system harvested at mungbean maturity. Values are means±standard error (n = 4). Values with different letters within a line indicate significant differences between treatment I_p_ and I_m_ (Tukey HSD, *p<0*.*05*).

The C content in the AGP of oats in the I_p_ and I_m_ treatments was 0.42 and 1.73 g plant^-1^, respectively. In comparison, the corresponding values of the roots were 0.16 and 0.51 g plant^-1^. In the oat AGP fraction, 0.03 and 0.45 g plant^-1^ C was transferred from mungbean in the I_p_ and I_m_ treatments, representing 8.2% and 24.5% of oat AGP-C, respectively. In addition, the ratio of transferred C from mungbean to oats in relation to total C in oats was 16.2% at pod setting and 22.0% at maturity (Table 6).

## Discussion

### Recovery of ^15^N and ^13^C

Less than 70% ^15^N was recovered from the S_p_, I_p_, and S_m_ treatments, which was lower than that obtained by similar studies conducted under controlled conditions, in which recovery rates of more than 80% were reported [29, 30, 34, 40]. However, this level was higher than that reported by previous studies conducted under field conditions, in which the recovery rates were reported to be about 60% [29].

The ^15^N that was not recovered in the plant-soil system might have been lost because of experimental errors in the mass balance between the calculated ^15^N added through labeling and its recovery from the various plant parts [34, 42], or loss as NH_3_ in urea solution from the wick system. The latter explanation was probably the most likely in this experiment. Specifically, at the first application, the ^15^N urea solution was taken up by the plants in less than 1 day, whereas the speed of uptake declined markedly during the second application, requiring at least 2 days. In addition, the recovery rates were higher at maturity than at pod setting, which might be attributed to the lower amount of ^15^N lost in the plant-soil system after pod setting.

Relatively higher amounts of applied ^13^C (more than 50%) were recovered in our study compared to that reported by similar field studies [29]. However, lower amounts of ^13^C were recovered compared to ^15^N, which might be partly attributed to the loss of assimilated ^13^C as CO_2_, due to either plant or microbial respiration [5, 36]. There are two possible explanations for the lower recovery of ^13^C at maturity than at pod setting in our study. First, the continued loss of ^13^C as CO_2_, supporting previous studies on pea and oat [29]. Second, the solution remained in the plant-soil system for a long time, resulting in an increase in the loss of the applied isotope by microorganisms. Therefore, short-term labeling has advantages over long-term labeling under field conditions, with the short time between labeling and sampling yielding better isotope recovery rates [13].

### Suitability of the ^15^N-^13^C double-labeling method in the intercropping system

To our knowledge, this is the first study in which the cotton-wick method was used for the ^15^N-^13^C double labeling of an intercropping system. This technology allowed the higher recovery of ^15^N and ^13^C and did not damage the root structure, which is a drawback of the split-root method [35]. The results of our study showed relatively higher and uniform isotope enrichment of mungbean plants, which was sufficient to trace the ^15^N and ^13^C in intercropped oat AGP and root fractions (Table 2 and 3). However, the plants in the intercropping system were not homogeneously labeled, with oat fractions (receiver) having lower enrichment than mungbean fractions. This difference may have been a consequence of higher enrichment closer to the label source, which influenced the distribution of the isotope in plants [14]. This trend has also been recorded in other ^15^N labeling experiments that used stem feeding [29, 34] and root labeling [19].

Furthermore, estimation of rhizodeposition by using Janzen and Bruisma’s equation needs to be performed carefully, particularly when the pulsed labeling method is used, because of the assumptions supporting the calculations [29, 43]. According to Mahieu et al. [44], rhizodeposition is overestimated for plants subject to the pulse-labeling technique compared to the continuous-labeling method. Labeling experiments lasting more than 1 year need be conducted, because short-term studies may be strongly affected by the weather and other uncontrollable factors.

### Quantity and partitioning of N and C

The NdfR (at both pod setting and maturity) was lower for mungbean than for peas labeled using similar methods [29, 31]. However, the percentage of NdfR at maturity was higher for mungbean than for peas, faba beans, and lupin that were labeled using a similar method [34], as well as for mungbean (17%) from a pot experiment performed using the petiole-feeding method [45], peas labeled using the split-root method [19, 21, 22], and alfalfa and soybean labeled under sterile conditions [46]. These differences indicate that the experimental conditions, labeling methods, and environment factors strongly influence NdfR estimates.

The NdfR was 3 times higher at the mature stage than at the pod stage. These results are consistent with those recorded for the pea (*Pisum sariuum* L.) by Jensen [19] using the split-root method, with lower NdfR at flowering than at maturity. However, our results contradicted those of Wichern et al. [29], who used a similar method. In addition, more NdfR was obtained in the intercropping system than in the sole cropping system, particularly at maturity. However, the dry matter of mungbean was not significantly different between the sole and intercropping system (Table 4). These results indicate that the N transferred to oats increases the NdfR of mungbean and might strengthen N-fixation. Furthermore, the ratio of NdfR to BGP-N in mungbean was similar to that reported in a previous study [13].

Overall, lower CdfR to total plant C was obtained for mungbean than for pea at both the pod and mature stages [29], but was higher than that for cereal and grasses reported in other studies [36, 47, 48]. This result might be attributed to the strong rhizodeposition ability of legumes compared to that of cereals, in addition to differences in the root construction and rhizospheric microorganisms of different plants. However, higher amounts of CdfR were obtained in the present study compared to those obtained by Wichern et al. [29] for peas and oats, despite the same method being used. A higher percentage of CdfR to total plant C was obtained in the mungbean-oat intercropping system than in the sole mungbean cropping system, under similar mungbean dry matter content (Table 4). This is because the transfer of C to intercropped oats enhanced the release of mungbean CdfR in the intercropping system.

### N and C transfer from labeled mungbean to oat plants

The labeling method used in the current study has been previously used to evaluate C and N rhizodeposition in field and laboratory studies [14, 29–31, 34, 40, 49, 50]. However, to our knowledge, this study is the first time the cotton-wick method was used to evaluate rhizodeposition and the transfer of C and N in a mungbean-oat intercropping system. The current study showed that N transfer represents more than 7% of total N in associated oats, particularly in the roots, which accounted for more than one-third of N transfer. Furthermore, the amount of N that was transferred to oats increased over time. Less N was transferred from mungbean to oats in this study than that reported in previous studies performed using the direct labeling method [51, 52] or other approaches [19]. For example, several experiments of intercropped barley and peas reported that 19% of N in barley was acquired from peas by using the split-root method [20]. However, other studies found little or no evidence of N transfer in legume-cereal intercropping systems [2, 49, 53, 54]. Thus, the mechanism of N transfer from legumes to cereal crops might be different, because of differences in plant photosynthesis intensity, root intermingling, and nodule activity.

In this study, mungbean contributed to 16.2% and 22.0% of total C uptake by oats at the pod setting and mature stages, respectively. The oat plants were at the filling stage when mungbean plants were mature. Thus, the strong nutritional need of oats at this stage led to a strong nutrient absorbing ability, which resulted in a higher proportion of C being present at harvest. Most previous studies have focused on the below-ground C of monocropping systems, and found part of the C was respired as CO_2_ incorporated into the soil organic matter or used by microbial [36]. However, these previous studies did not investigate the transfer of below-ground C in intercropping systems, even though the amount was comparatively low. Our result show that mungbean contributes to about 20% of the C in intercropped oat, and that this part of C cannot be easily neglect. The increased yield of non-legumes might be partly attributed to the amount of C transferred. Nevertheless, information about the importance of C transfer from legumes to non-legumes remains limited, we cannot easily coming to a conclusion.

Although our study provides new insights into the effects of intercropping mungbean with oats on NdfR and CdfR, as well as the transfer of N and C from mungbean to associated oats, the exact mechanisms that influence the amount and quality of NdfR and CdfR during intercropping remain unknown. Moreover, considerable research is required to determine the dynamics of NdfR and CdfR during the plant growth period. Subsequent research should focus on the influence of intercropping on the release of NdfR and CdfR into different soil pools and their utilization by successive crops, by quantifying the nutritional benefit provided to subsequent crops in rotation. Other parameters that warrant further studies include the influence of genotypic variation on rhizodeposition in important crops, N and C transfer from plants to soil microorganisms, and the role of rhizodeposition on C sequestration and N nutrition.

## Conclusions

The mungbean and oat intercropping system can be efficiently labeled and studied using the *in situ* ^15^N-^13^C double stem-feeding method under field conditions, although maybe short term duration is one of the problems. This study showed that mungbean NdfR contributes to more than half of BGP-N, whereas CdfR accounts for about 60% of BGP-C. In addition, intercropping and time extension can increase the NdfR and CdfR of mungbean. Furthermore, significant amounts of BGP-N were transferred from mungbean to oats in the intercropping system under field conditions, accounting for about 10% total N of associated oats, whereas C transfer accounted for about 20%. It shows the C transferred from mungbean to oats in the intercropping system should not be overlooked. The results of this study show that only a small proportion of rhizodeposition from mungbean is transferred to oats under the intercropping system, with a large portion remaining in the soil. This finding implies that mungbean rhizodeposition could represent a potentially large source of nutrients in the cropping systems of northeast China.

## Supporting Information

S1 Table ^15^N and ^13^C nature enrichment (‰) in the plant and soil parts. (DOCX)

## References

[1] Francis CA. Biological efficiencies in multiple-cropping systems. Adv Agron. 1989;42: 1–42.

[2] Xiao Y, Li L, Zhang F. Effect of root contact on interspecific competition and N transfer between wheat and fababean using direct and indirect ^15^N techniques. Plant Soil. 2004;262: 45–54.

[3] Peoples M, Boddey R, Herridge D, Leigh G. Quantification of nitrogen fixation In: Leigh GJ, editor. Nitrogen fixation at the millennium. Elsevier, Brighton, UK 2002 pp. 357–389.

[4] Whipps JM. Carbon economy In: Lynch JM, editor. The rhizosphere. Wiley, Chicheater 1990 pp. 59–97.

[5] Nguyen C. Rhizodeposition of organic C by plants: mechanisms and controls. Agronomie. 2003;23: 375–396.

[6] Rovira A. Plant root excretions in relation to the rhizosphere effect. Plant Soil. 1956;7: 178–194.

[7] Gregory P. Roots, rhizosphere and soil: the route to a better understanding of soil science? Euro J Soil Sci. 2006;57: 2–12.

[8] Marschner H. Mineral nutrition of higher plants. London: Academic Press 1995.

[9] Hütsch BW, Augustin J, Merbach W. Plant rhizodeposition—an important source for carbon turnover in soils. J Plant Nut Soil Sci. 2002;165: 397–407.

[10] Chalk P, Ladha J, Padre A. Efficacy of three ^15^N labelling techniques for estimating below-ground N in Sesbania rostrata. Biol Fert Soils. 2002;35: 387–389.

[11] De Graaff MA, Six J, Van Kessel C. Elevated CO_2_ increases nitrogen rhizodeposition and microbial immobilization of root‐derived nitrogen. New Phytol. 2007;173: 778–786. 17286826 10.1111/j.1469-8137.2006.01974.x

[12] Ledgard S, Freney J, Simpson J. Assessing nitrogen transfer from legumes to associated grasses. Soil Biol Biochem. 1985;17: 575–577.

[13] Khan D, Peoples M, Herridge D. Quantifying below-ground nitrogen of legumes. 1. Optimising procedures for ^15^N shoot-labelling. Plant Soil. 2002;245: 327–334.

[14] Yasmin K, Cadisch G, Baggs E. Comparing ^15^N-labelling techniques for enriching above-and below-ground components of the plant-soil system. Soil Biol Biochem. 2006;38: 397–400.

[15] Götz K, Herzog H. Distribution and utilization of ^15^N in cowpeas injected into the stem under influence of water deficit. Isot Environ Healt S. 2000;36: 111–121. 10.1080/10256010008032937 11077926

[16] Janzen H. Deposition of nitrogen into the rhizosphere by wheat roots. Soil Biol Biochem. 1990;22: 1155–1160.

[17] McNeill A, Hood R, Wood M. Direct measurement of nitrogen fixation by Trifolium repens L. and Alnus glutinosa L. using ^15^N_2_ . J Exp Bot. 1994;45: 749–755.

[18] Merbach W, Schulze J, Richert M, Rrocco E, Mengel K. A comparison of different ^15^N application techniques to study the N net rhizodeposition in the plant‐soil system. J Plant Nut Soil Sci. 2000;163: 375–379.

[19] Jensen E. Rhizodeposition of N by pea and barley and its effect on soil N dynamics. Soil Biol Biochem. 1996;28: 65–71.

[20] Jensen E. Barley uptake of N deposited in the rhizosphere of associated field pea. Soil Biol Biochem. 1996;28: 159–168.

[21] Sawatsky N, Soper R. A quantitative measurement of the nitrogen loss from the root system of field peas (Pisum avense L.) grown in the soil. Soil Biol Biochem. 1991;23: 255–259.

[22] Schmidtke K. How to calculate nitrogen rhizodeposition: a case study in estimating N rhizodeposition in the pea (Pisum sativum L.) and grasspea (Lathyrus sativus L.) using a continuous ^15^N labelling split-root technique. Soil Biol Biochem. 2005;37: 1893–1897.

[23] Kuzyakov Y, Kretzschmar A, Stahr K. Contribution of Lolium perenne rhizodeposition to carbon turnover of pasture soil. Plant Soil. 1999;213: 127–136.

[24] Warembourg FR, Estelrich HD. Towards a better understanding of carbon flow in the rhizosphere: a time-dependent approach using carbon-14. Biol Fert soils. 2000;30: 528–534.

[25] Domanski G, Kuzyakov Y, Siniakina S, Stahr K. Carbon flows in the rhizosphere of ryegrass (Lolium perenne). J Plant Nut Soil Sci. 2001;164: 381.

[26] Kuzyakov Y, Cheng W. Photosynthesis controls of rhizosphere respiration and organic matter decomposition. Soil Biol Biochem. 2001;33: 1915–1925.

[27] Schenck zu Schweinsberg‐Mickan M, Jörgensen RG, Müller T. Rhizodeposition: Its contribution to microbial growth and carbon and nitrogen turnover within the rhizosphere. J Plant Nut Soil Sci. 2012;175: 750–760.

[28] Merbach W, Mirus E, Knof G, Remus R, Ruppel S, Russow R, et al Release of carbon and nitrogen compounds by plant roots and their possible ecological importance. J Plant Nut Soil Sci. 1999;162: 373–383.

[29] Wichern F, Mayer J, Joergensen R. Rhizodeposition of C and N in peas and oats after ^13^C-^15^N double labelling under field conditions. Soil Biol Biochem. 2007;39: 2527–2537.

[30] Russell C, Fillery I. In situ ^15^N labelling of lupin below-ground biomass. Aust J Agr Res. 1996;47: 1035–1046.

[31] Wichern F, Mayer J, Joergensen RG, Müller T. Release of C and N from roots of peas and oats and their availability to soil microorganisms. Soil Biol Biochem. 2007;39: 2829–2839.

[32] Yasmin K, Cadisch G, Baggs E. The significance of below-ground fractions when considering N and C partitioning within chickpea (Cicer arietinum L.). Plant Soil. 2010;327: 247–259.

[33] Laberge G, Haussmann BI, Ambus P, Høgh-Jensen H. Cowpea N rhizodeposition and its below-ground transfer to a co-existing and to a subsequent millet crop on a sandy soil of the Sudano-Sahelian eco-zone. Plant Soil. 2011;340: 369–382.

[34] Mayer J, Buegger F, Jensen ES, Schloter M, Heß J. Estimating N rhizodeposition of grain legumes using a ^15^N in situ stem labelling method. Soil Biol Biochem. 2003;35: 21–28.

[35] Mahieu S, Fustec J, Faure M-L, Corre-Hellou G, Crozat Y. Comparison of two ^15^N labelling methods for assessing nitrogen rhizodeposition of pea. Plant Soil. 2007;295: 193–205.

[36] Kuzyakov Y, Domanski G. Carbon input by plants into the soil. Review. J Plant Nut Soil Sci. 2000;163: 421–431.

[37] Jones D, Nguyen C, Finlay R. Carbon flow in the rhizosphere: carbon trading at the soil–root interface. Plant Soil. 2009;321: 5–33.

[38] Li M, Hu YG, Zeng ZH, Ren CZ, Mao N, Song WJ, et al Wind tunnel experiment on anti-wind erosion capacity of four crop stubbles in horqin sandy land. Chinese Agricultural Science Bulletin. 2009;11: 053.

[39] Yang XC. Effect of Nitrogen Fertilizer and Rhizodeposition of Oat Intercropped with Mung bean. M.Sc. Thesis, China Agricultural University. 2012

[40] Russell C, Fillery I. Estimates of lupin below-ground biomass nitrogen, dry matter, and nitrogen turnover to wheat. Aust J Agr Res. 1996;47: 1047–1059.

[41] Janzen H, Bruinsma Y. Methodology for the quantification of root and rhizosphere nitrogen dynamics by exposure of shoots to ^15^N-labelled ammonia. Soil Biol Biochem. 1989;21: 189–196.

[42] Rroço E, Mengel K. Nitrogen losses from entire plants of spring wheat (Triticum aestivum) from tillering to maturation. Eur J Agron. 2000;13: 101–110.

[43] Rasmussen J. Why we need to restrict the use of “rhizodeposition” and the Janzen and Bruinsma equation. Soil Biol Biochem. 2011;43: 2213–2214.

[44] Mahieu S, Fustec J, Jensen ES, Crozat Y. Does labelling frequency affect N rhizodeposition assessment using the cotton-wick method? Soil Biol Biochem. 2009;41: 2236–2243.

[45] Khan D, Peoples M, Chalk P, Herridge D. Quantifying below-ground nitrogen of legumes. 2. A comparison of ^15^N and non isotopic methods. Plant Soil. 2002;239: 277–289.

[46] Brophy LS, Heichel G. Nitrogen release from roots of alfalfa and soybean grown in sand culture. Plant Soil. 1989;116: 77–84.

[47] Helal H, Sauerbeck D. Influence of plant roots on C and P metabolism in soil. Plant Soil. 1984;76: 175–182.

[48] Kuzyakov Y, Schneckenberger K. Review of estimation of plant rhizodeposition and their contribution to soil organic matter formation. Arch Agron Soil Sci. 2004;50: 115–132.

[49] Hertenberger G, Wanek W. Evaluation of methods to measure differential ^15^N labeling of soil and root N pools for studies of root exudation. Rapid Commun Mass Sp. 2004;18: 2415–2425. 15386635 10.1002/rcm.1615

[50] Jamont M, Piva G, Fustec J. Sharing N resources in the early growth of rapeseed intercropped with faba bean: does N transfer matter? Plant Soil. 2013;371: 641–653.

[51] Høgh-Jensen H, Schjoerring JK. Below-ground nitrogen transfer between different grassland species: Direct quantification by ^15^N leaf feeding compared with indirect dilution of soil ^15^N. Plant Soil. 2000;227: 171–183.

[52] Chu G, Shen Q, Cao J. Nitrogen fixation and N transfer from peanut to rice cultivated in aerobic soil in an intercropping system and its effect on soil N fertility. Plant Soil. 2004;263: 17–27.

[53] Danso S, Zapata F, Hardarson G, Fried M. Nitrogen fixation in fababeans as affected by plant population density in sole or intercropped systems with barley. Soil Biol Biochem. 1987;19: 411–415.

[54] Izaurralde R, McGill W, Juma N. Nitrogen fixation efficiency, interspecies N transfer, and root growth in barley-field pea intercrop on a Black Chernozemic soil. Biol Fert Soils. 1992;13: 11–16.

