# Correction: Rhizodeposition of Nitrogen and Carbon by Mungbean (*Vigna radiata* L.) and Its Contribution to Intercropped Oats (*Avena nuda* L.)

**DOI:** 10.1371/journal.pone.0128503

**Published:** 2015-05-20

**Authors:** The PLOS ONE Staff

Table 2 of the published article is incorrect. Please view the correct Table 2 here.

**Table 2:** Atom% ^15^N excess, recovery and distribution of ^15^N in each part of plant and soil in sole cropped and intercropped systems.

		Mungbean				Oat			
		Grain	Stem	Leaves	Roots	APG ^1^	Roots	Rhizodeposition	Total
Atom% ^15^N excess	S_p_	0.11±0.05^a^	0.20±0.08^a^	0.10±0.04^a^	0.09±0.05^a^	-	-	-	-
	I_p_	0.13±0.09^a^	0.24±0.12^a^	0.08±0.02^a^	0.10±0.08^a^	0.001±0.000^b^	0.02±0.00^a^	-	-
	S_m_	0.07±0.03^a^	0.16±0.07^a^	0.07±0.04^a^	0.08±0.06^a^	-	-	-	-
	I_m_	0.09±0.06^a^	0.18±0.12^a^	0.11±0.01^a^	0.05±0.03^a^	0.002±0.000^a^	0.02±0.00^a^	-	-
Recovery of ^15^N (in % of applied)	S_p_	7.98±4.42^b^	20.48±5.90^a^	26.08±8.59^a^	3.26±1.19^a^	-	-	4.16±1.35^a^	61.96±14.42^a^
	I_p_	18.73±17.23^ab^	24.29±7.76^a^	17.56±2.26^ab^	4.20±3.40^a^	0.02±0.01^a^	0.09±0.00^b^	3.51±1.33^a^	68.41±25.58^a^
	S_m_	35.86±10.86^ab^	10.04±4.34^a^	7.57±3.76^b^	4.07±2.27^a^	-	-	5.72±2.73^a^	63.26±13.24^a^
	I_m_	50.74±19.42^a^	12.33±6.65^a^	11.45±5.96^b^	1.40±0.32^a^	0.18±0.05^a^	0.28±0.23^a^	5.44±0.47^a^	81.87±23.02^a^
Distribution of recovered ^15^N (%)	S_p_	12.92±6.62^b^	33.03±4.44^b^	41.42±8.19^a^	5.34±1.51^a^	-	-	7.27±3.50^a^	100
	I_p_	23.74±13.50^b^	36.14±4.47^b^	27.31±6.96^ab^	6.69±6.07^a^	0.04±0.02^b^	0.14±0.04^a^	5.94±3.22^a^	100
	S_m_	56.06±6.38^a^	16.33±8.48^a^	11.57±3.56^c^	6.22±2.67^a^	-	-	9.82±5.70^a^	100
	I_m_	61.75±13.68^a^	15.35±8.91^a^	13.53±5.66^bc^	1.78±0.37^a^	0.22±0.02^a^	0.31±0.21^a^	7.06±2.34^a^	100

^1^ Above-ground part

Mungbean were labeled five weeks after planting and harvested at the beginning of pod setting and at maturity. Mungbean were divided into grain, stem, leaves and root, while oats were divided into above-ground part and root, and rhizodeposition.

The abbreviations in the table represent different treatments. S_p_: sole mungbean harvested at pod setting, S_m_: sole mungbean harvested at maturity, I_p_: intercropping system harvested at mungbean pod setting, and I_m_: intercropping system harvested at mungbean maturity. Values are means±standard error (n = 4). Values with different letters within a column indicate significant differences between the treatment S_p_, I_p_, S_m_ and I_m_ (Tukey HSD, *p<0*.*05*).
