# Supplementary material for: Rhizodeposition of Nitrogen and Carbon by Mungbean (Vigna radiata L.) and Its Contribution to Intercropped Oats (Avena nuda L.)
Source: PLoS One. 2015 Mar 30;10(3):e0121132. doi: 10.1371/journal.pone.0121132 (PMC4379154; doi:10.1371/journal.pone.0121132)
Supplement: S1 Table — (DOCX) [file pone.0121132.s001.docx]

**S1 Table. ^15^N and ^13^C nature enrichment (‰) in the plant and soil parts.**

|  | **^15^N** | **^13^C** |
| --- | --- | --- |
| **Mungbean** |  |  |
| Grain | 3.68 | 10.79 |
| Stem | 3.68 | 10.77 |
| Leaves | 3.69 | 10.78 |
| Roots | 3.70 | 10.77 |
| **Oat** |  |  |
| AGP ^1^ | 3.70 | 10.78 |
| Roots | 3.69 | 10.76 |
| **Soil** | 3.69 | 10.81 |

^1^ Above-ground part
